# New Approach for the Development of Improved Traditional Medicine: Case of a Preparation of an Oral Hypoglycemic Medicine from *Laportea ovalifolia* (Schumach. & Thonn.) Chew. (Urticaceae)

**DOI:** 10.4172/2329-9053.1000125

**Published:** 2015-07-22

**Authors:** Nolé Tsabang, Stella Kadjob, Rose N. Mballa, Clement G. Yedjou, Nga Nnanga, Néhémie T. Donfagsiteli, Alembert T. Tchinda, Gabriel A. Agbor, Claudine Ntsama, Paul B. Tchounwou

**Affiliations:** 1 Center for Research on Medicinal Plants and Traditional Medicine, Institute of Medical Research and Medicinal Plants Studies (IMPM), Ministry of Scientific Research and Innovation, Yaoundé, Cameroon; 2 High Institute of Environmental Sciences, Yaoundé, Cameroon; 3 Faculty of Medicine and Biomedical Sciences, Yaoundé, Cameroon; 4 Cellomics and Toxicogenomics Research Laboratory, NIH-RCMI Center for Environmental Health, Jackson State University, Jackson, USA; 5 Laboratory of Cellular Signalling, Phytoceuticals, Cancer Prevention and Therapies, College of Science, Engineering and Technology, Jackson State University, Jackson, USA

**Keywords:** Improved traditional medicine, New production strategy, Oral hypoglycemic phytodrug, Sustainable exploitation

## Abstract

A majority of Africans rely on traditional medicine as the primary form of health care. Yet most traditional medicine products have a short shelf life, especially for water-based formulations such as macerations, infusions and decoctions. Indeed, many of these water extracts become unfit for human consumption after five to seven days of conservation either because of the degradation or toxicity of active components, and/or the growth of pathogenic organisms. The purpose of this study was to describe and apply a new approach for the development of an improved traditional medicine (ITM) that is cheap, very efficient, not toxic, and easy to produce, and that can be conserved for a longer time without a significant loss of activity. Hence, *Laportea ovalifolia* was selected from an ethnobotanical prospection in all regions of Cameroon, and was used to prepare an oral hypoglycemic product. This preparation required 9 steps focused on the characterization of the plant species, and the standardization of the ethnopharmacological preparation by a multidisciplinary team of scientists with expertise in botany, ecology, pharmacognosy and pharmacology. Resultantly, four galenic formulations of hypoglycemic medications were produced. A relationship between these four formulations was described as follow: One spoon of oral suspension (10 ml)=one sachet of powder=2 tablets=3 capsules. Hence, our research provides new insight into a drug discovery approach that could alleviate the major problems affecting traditional medicine and enhance its effectiveness in addressing health care in developing and undeveloped countries.

## Introduction

Biodiversity is the foundation of human survival, and economic well-being. It also constitutes the bioresources upon which individuals, families, communities, nations and future generations depend [1]. The existing knowledge of ethnic medicines has developed several leads in health care and drug discovery, and as a pattern for discovery [2]. Traditional, complementary and alternative medicines attract the full spectrum of reactions. Yet, the use of traditional, complementary and alternative medicine is increasing rapidly in developing countries. In many parts of the world, policy-makers, health professionals and the public are wrestling with questions about the safety, efficacy, quality, availability, preservation and further development of this type of health care [3].

The Institute of Medical Research and Medicinal Plants Studies and different Faculties of Medicine and Biomedical Sciences in Cameroon, encourage strongly innovated initiatives in the field of development of ITM. Many medicinal plants including Prunus afrinana, Pausinystalia yoimbe, Morinda lucida, Laportea ovalifolia, Momordica charantia, Catharanthus roseus and Moringa oleiracea are commonly used in traditional medicine [4]. Such plants with established clinical effects have been actively studied in laboratories and some of them exported. Meanwhile, almost 90% of African countries which suffer from biodiversity erosion and climate changes, and emergence and reemergence of human diseases, depend on foreign pharmaceutical industries and laboratories, and therefore are in great need of new ITM from these medicinal plants. *Laportea ovalifolia* is used in traditional medicine in Cameroon to treat diabetes [5,6]. Previous studies with diabetic rats have shown that this plant species is non-toxic, and produces hypoglycemic activities characterized by significant decreases of glucose concentrations in the blood [7,8].

Most of African countries lack technological and scientific capabilities to participate in commercial collaborations and opportunities created by Biodiversity Convention. Furthermore, because of the lack of financial resources and infrastructures to reduce the burden of diseases, African researchers have limited expertise and poor access to the necessary technological infrastructure for research discovery on phytodrugs. Many countries manifestly have a need to develop strategies to carry out botanical bio prospection for producing new medicines and other products. Africa is in good position to invest its energy for more focused solutions that can bring tangible economic improvements to subsistence means of localities.

The World Health Organization (WHO) recognizes that in Africa, about 80% of the population use traditional medicine to address their health needs. The Libreville’s WHO/OAPI initiative on valorization of traditional medicine has launched, a referential document for registration of phytomedicines which defines categories 1, 2, 3 and 4 [9]. In a parallel direction, this document notes that medicinal plants constitute one of the action areas of this initiative. In connection to that line of thinking, Adjanohoun (1982) pointed out that «Throughout Black Africa, the great majority of medicinal plants are still used in crude state in the forms of extracts». In addition to the knowledge of these simple traditional formulations, it is important to know the dose of active ingredients, and to control of the side effects and/or toxicity; both processes of which are not perfectly mastered by traditional healers.

For above-stated reasons the present study proposes a necessary methodology of investigations to develop a new ITM that is cheaper, very efficient, not toxic and easily produced to treat several pathologies that affect more undeveloped countries. The proposed methodology focuses on the simplification of the necessary technology of drug manufacturing and obeys to the conditions of rational plant harvest, conservation of plant materials harvested, and production of the final drugs. The selection of the species used amongst many plants requires a particular attention. Many other challenges to be achieved in drug discovery are the identifying of the plants’ mechanisms of action, the respect of the clearance ethic, the intellectual property rights (the sharing of the benefice or the income generated by the product) and the legal procedure [3]. The use of this methodology can stimulate a sustainable development by providing the basis for drugs discovery and by documenting biodiversity for long time exploitation.

## Materials and Methods

### Ethnobotanical participative survey

*Laportea ovalifolia* has been used medicinally and nutritionally in numerous traditions of folk healing among many indigenous cultures of Cameroon. Therefore to select this plant among others, we conducted a survey in all ecological zones of Cameroon. Folklore medicinal information on antidiabetic medicinal plants were recorded through discussions with 1131 interviewees including traditional healers, knowledgeable elder people and householders, using a semi-structured questionnaire that included the village name, name of respondent, scientific and common names of medicinal plants, and conditions (fresh or dry) of the plant parts. Each of the plant species collected with the help of informants was recorded, and botanic samples were identified using the existing standard floras and specimens. The voucher specimens were deposited in the National Herbarium of Cameroon. *Laportea ovalifolia* was the most credible antidiabetic plant recorded [6] for ethno pharmacological preparation of ITM.

### Characterization of *Laportea ovalifolia*

The development of new ITM from *Laportea ovalifolia* required 9 steps focused on the following sciences: Botany, Ecology, Ethnobotany, Ethnomedicine, Ethnopharmacology, Pharmacology, Conservation, Pharmacognosy and Pharmacy. These sciences have intervened in the characterization of the species and the standardization of the ethno pharmacological preparation.

### Ecology

*Laportea ovalifolia* reproduces by seeds and grows in shady environments. It is widely spread in humid tropical African regions, from altitudes 0 to 2800 m, and from Sierra Leone to Uganda and Angola.

### Botany

*Laportea ovalifolia* is a dioecious herbaceous weed that is more often creeping than erect. Male L. ovalifolia has big leaves while the female has small leaves. The variety harvested is a perennial male plant, with cylindrical stems 0.50 to 1.20 cm high; alternate petiolate leaves, 8 to 15 cm long and 4 to 8 cm wide and lanceolate limb. All the morphological parts of the two varieties are densely covered with stinging scattered brown hairs and perennial stems are cylindrical, greenish and sometimes reddish or brownish.

### Pharmacognosy

The previous pharmacological studies on *Laportea ovalifolia* were focused on identifying the active extracts and/or compounds, assessing hypoglycemic proprieties, and drug safety, conducting clinical trials, and determining the mechanisms of action such as peripheral or extra pancreatic action, insulin inactivation, increased permeability of cells membranes, oxidation-reduction reactions, inhibition or reduction of the intestinal glucose absorption, regeneration/revitalization of residual ß-cells, and hormonal glucose regulation [10].

### Phytochemistry and pharmacology

Broad range of chemical constituents has been separated from *Laportea ovalifolia* extracts. These include abundant saponins, sodium bicarbonate and cardiac glycosides; moderate amount of tannins, flavonoids, polyphenols and sterols, and trace amount of alkaloids, in water, ethanol, hexane and methanol extracts. Pharmacological hypoglycemic activities have been reported from tests with the crude extracts of this plant, as well as in experiments using isolated components such as glycosides, sterols and flavonoids [5]. Therefore this herb possesses a number of active ingredients which contribute to its effectiveness as an antidiabetic folk medicine. The extract in the equal volume of methylene chloride and methanol contains more compounds [5]. The toxicity of methanol extract is currently known. Complete toxicity study that was conducted on this plant revealed the safety of its extracts [5]. In addition to the hypoglycemic mechanisms of action reported above, *Laportea ovalifolia* may also stimulate the remnant beta cells and enhance glucose utilization by peripheral tissues [5].

### Preparation of the standard extract

Identification of the recipe: A field survey in five phytogeographic regions of Cameroon offered the opportunity to record many hypoglycemic recipes from *Laportea ovalifolia* produced by 246 traditional healers. It was observed that the traditional healer from Fongo-Tongo (West region of Cameroon) used by her formulation to treat 30 diabetic patients who were previously diagnosed at the local health center. This recipe proved to be very effective in reducing the blood glucose concentrations in patients with type 2 diabetes (Table 1).

Therefore, the aqueous extract of this formulation was replicated in the Botany and Traditional Medicine’ laboratory at the Institute of Medical Research and Medicinal Plants Studies in Yaounde (Camroon), following the successful mode of preparation (decoction), with care taken to apply hygienic rules.

### Ethno pharmacological preparation and ethnomedical administration of the standard recipe

The ethnopharmacological preparation and the ethnomedical administration of the standard extract were described with the accurate description of the quantity of plant material, the volume of solvent used, the boiling temperature and the duration of preparation, the route of administration, the dose of medicine used per day, the duration of treatment (number of days), the secondary and undesirable effect(s) and the associated diets.

### Description of the standard recipe

One hundred g of leafy stems of *Laportea ovalifolia* were boiled at 100°C in 6 liters of distilled water for 25 min. For diabetes treatment, each patient was then asked to drink a glass of the extract (250 ml) in the morning, mid-day and evening, for 10 days. No secondary and undesirable effects were recorded.

### Pharmacy

Determination of the dose: This dose is generally one glass (250 ml). To identify the standard dose, 10 glasses of 250 ml each of the standard extract were lyophilized independently and the mean weight of dry powder obtained constituted the therapeutic dose. This dose is equivalent to the traditional healer minimal extract dose administrated successfully one time to a given patient, during the treatment. We have determined a percentage of the lyophilized powder which is the quantity of powder in 100 ml of distilled water. According to the therapeutic dictionary [11], the therapeutic dose obtained, shared in two tablets, was added to a determined quantity of excipients. Considering the content of a capsule, being 0.205 g the quantity of therapeutic dose and excipients for each of the three capsules was also determined [11]. Finally, two tablets, three capsules, one powder in sachet and a bottle of 125 ml were prepared.

### Good management of intellectual property rights of indigenous knowledge

We have respected the intellectual property rights in the development of this hypoglycemic ITM. So the income generated from selling this ITM will alleviate the poor traditional healer’ family and its community. It will help public and my private research, according to Diamond Chakraborty ruling of United State Supreme Court [12].

## Results

### Preparation and formulation of the four galenic forms of the drugs (oral suspension, capsules, powder in sachets and tables)

Oral suspension: The quantity of the lyophilized powder per therapeutic dose/equivalent of a 10 ml spoon was 0.525 g. In 10 ml of distilled water this quantity of lyophilized powder was dissolved. The quantity of powder in 100 ml was the percentage. Therefore the quantity of lyophilized powder for a therapeutic conditioning of a bottle of 125 ml was 6.5625 g and the treatment regimen was 10 ml × 3 per day before food for 10 days.

### Powder in sachets

The therapeutic dose is conserved in a sachet and one sachet was taking one time mixed with water. The quantity of lyophilized powder per sachet was 0.525 g and the treatment regimen was 1 sachet × 3 per day (before food) for 10 days.

### Tablet

The different excipients per a tablet were 10% of inert maize starch=0.003322785 g; 8% of monohydrated lactose=0.265823 g); 1.5% Talc=0.0049842 g and 1.5% Magnesium of stearate=0.0046842 g. These excipients were mixed with 79% of the lyophilized powder=262.5 mg. The theoretical weight of a tablet was calculated as follow p=0.2625 g × 100/79=0.3322785 g. The weight of a tablet 0.340 g was a mean of 10 tablets’ weight. The posology was 2 tablets × 3/day before food, for 10 days.

### Gelules

The content of one capsule was 0.205 g. We have mixed 0.030 g of inert maize starch and 0.175 g of lyophilized powder that represent respectively 14.634% and 85.366% of a capsule weight. The treatment regimen was 3 capsules × 3 per day before food for 10 days.

### Characteristics of the ITM

#### Relationship between the four forms

We prepare four galenic forms with relationship between them as follow: One spoon of oral suspension (10 ml)=one sachet of powder=2 tablets=3 capsules.

#### Indication

This ITM was prepared for type 2 diabetes or non-insulin dependent diabetes. It is to be used in association with an appropriate diet. In case of transitory glycemic unbalance, a short period of administration of product can be sufficient in a patient.

#### Contra-indications

Do not use this ITM in patients with insulin dependent diabetes or juvenile diabetes, ketoacidosis diabetes, pre-coma diabetes, chronic kidney disease and hepatic insufficiency.

#### Precautions

The following precautions are required: check the fasting glycemic level on a regular basis, control the diet, and perform physical exercise regularly.

## Discussion

The ITM new approach developed above represents an important work, and this accomplishment deserves continued recognition. Meanwhile, diabetes is an exceptionally difficult disease to manage. So for the individual it requires a lifelong commitment to dietary change, physical exercise, self-monitoring and medication regime (oral or injectable) and even dialysis. It is why our ITM is indicated for uncomplicated diabetes. The medicinal plants as an essential element of health care, are still widely used and have considerable importance in the national and international trade. The knowledge of their clinical, pharmacological and economical value continues to increase, though this varies strongly according to countries [13,14]. In the Republic of Mali the incidences of ITM in percentage are respectively 1,57; 1,64; 2,02 for the Community Health Center of Katiele and 1,69, 3,18, 2,71 for the Community Kebeni in 2001; 2002 and 2003 [15]. The multicomponent principle of our ITM, which aims were to sustain the reduction of the glucose rate in diabetics which take the traditional recipe will consequently allow us to predict more effective synergistic compounds. Like in the case of Traditional Oriental Medicines (TOMs), we anticipate that the identification of active compounds of our ITM and their effective targets will remain a challenge for some years [16]. The results of searches realized on Vernonia kotschyana roots are a contribution to the valorization of a recipe from traditional medicine in Mali, following five steps [17]. To minimize the cost of our ITM, we choose a plant for which many previous studies have been done on its different extracts; including toxicity tests for confirming its safety; phytochemical analysis for a better knowledge of its constituents, and pharmacological activity study to confirm it therapeutic anti-diabetic property. Others steps performed in the development of Vernonia kotschyana roots including microscopic study of the stem powder, clinical trials, and study of the market, have not yet been done for our ITM. While waiting to conduct additional studies, our immediate next step would be to perform clinical trials to further validate the effectiveness and national utilization of our ITM for the treatment of Type 2 diabetes.

**Figure 1:**
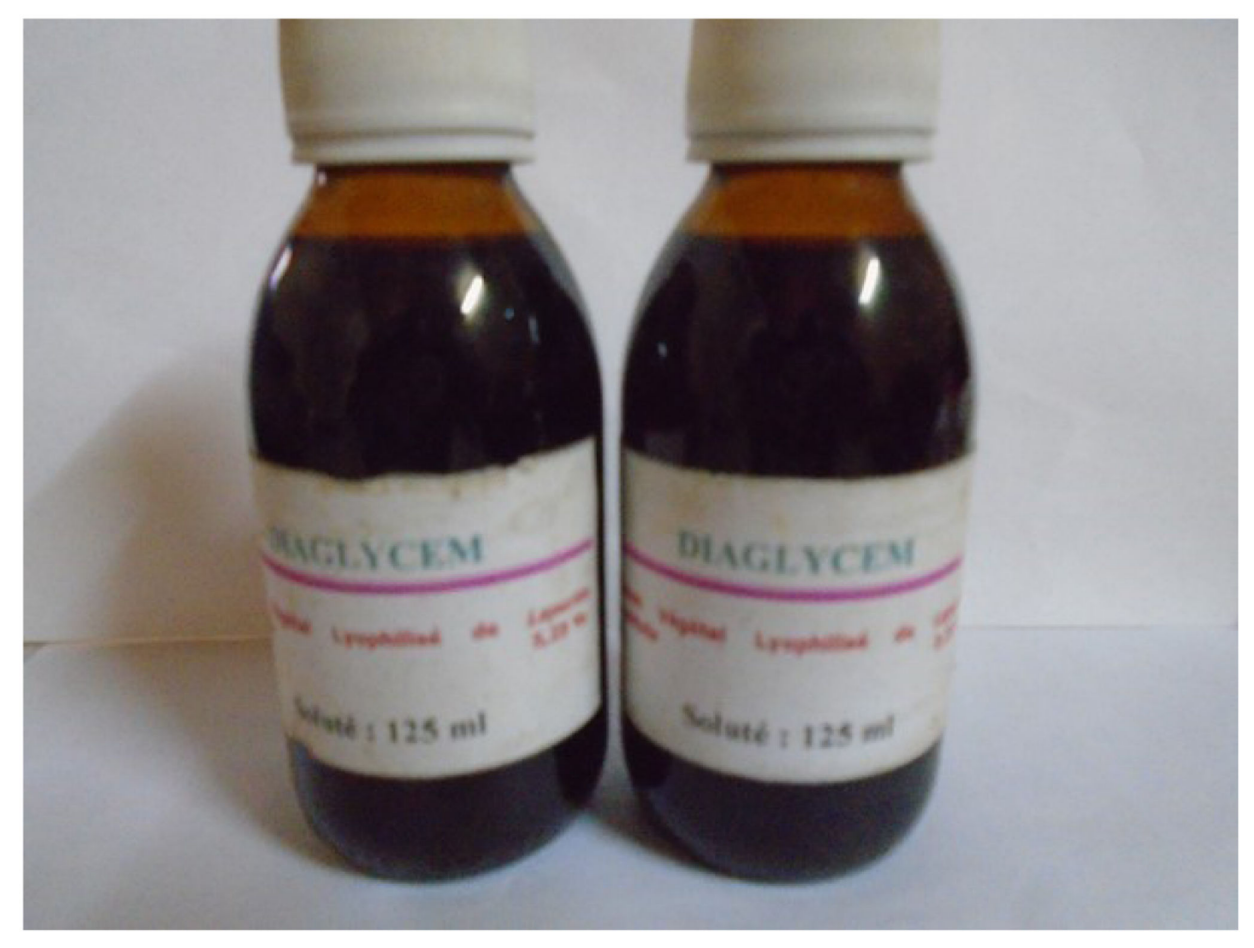
Oral suspension (This ITM was prepared in 2007 and this photograph was taken in June 2015 (no oxidation, no deposit at the bottom of the bottle)).

**Figure 2:**
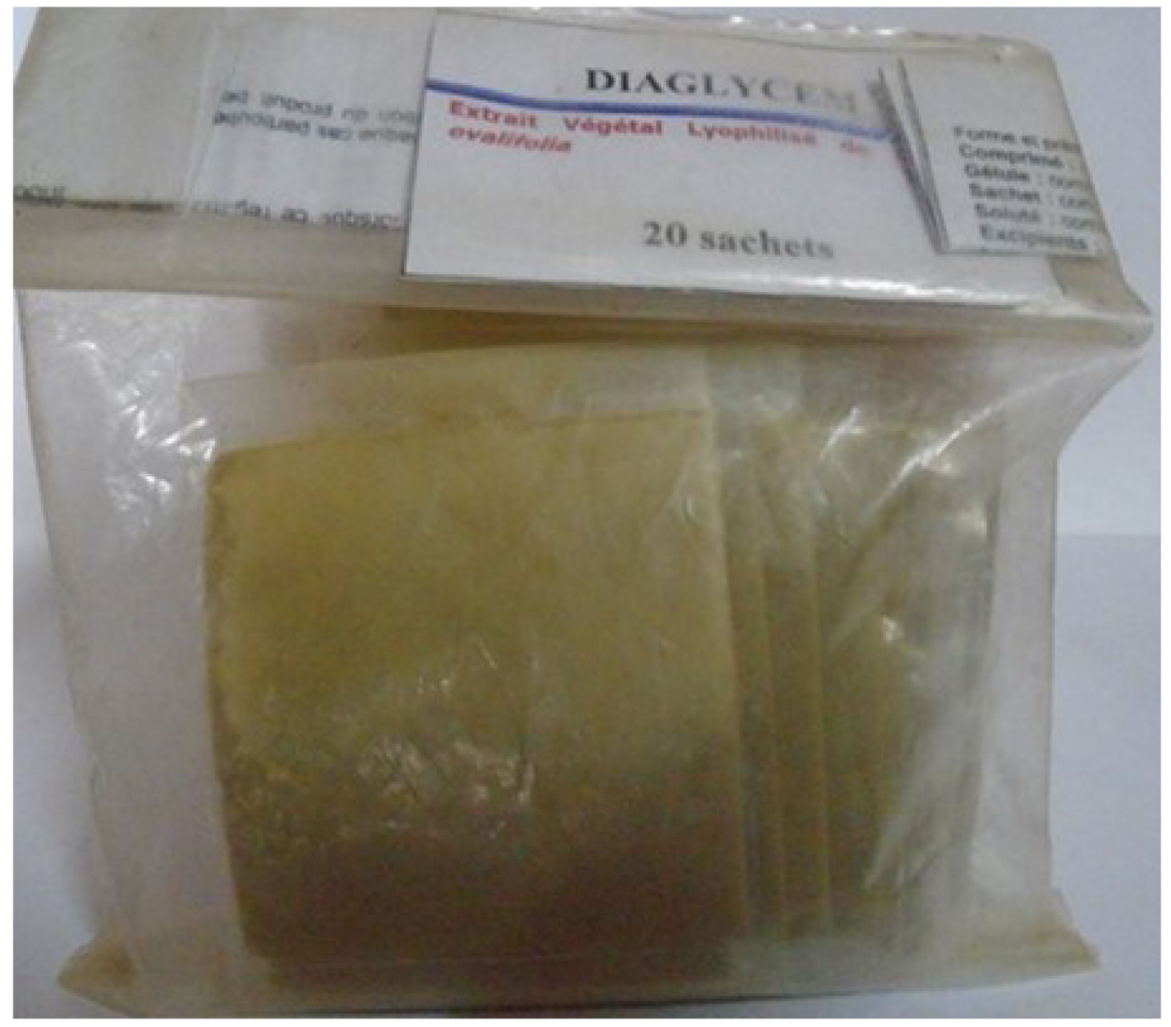
Powder in sachets (This ITM was prepared in 2007 and this photograph was taken in June 2015 (no deterioration observed until now)).

**Figure 3:**
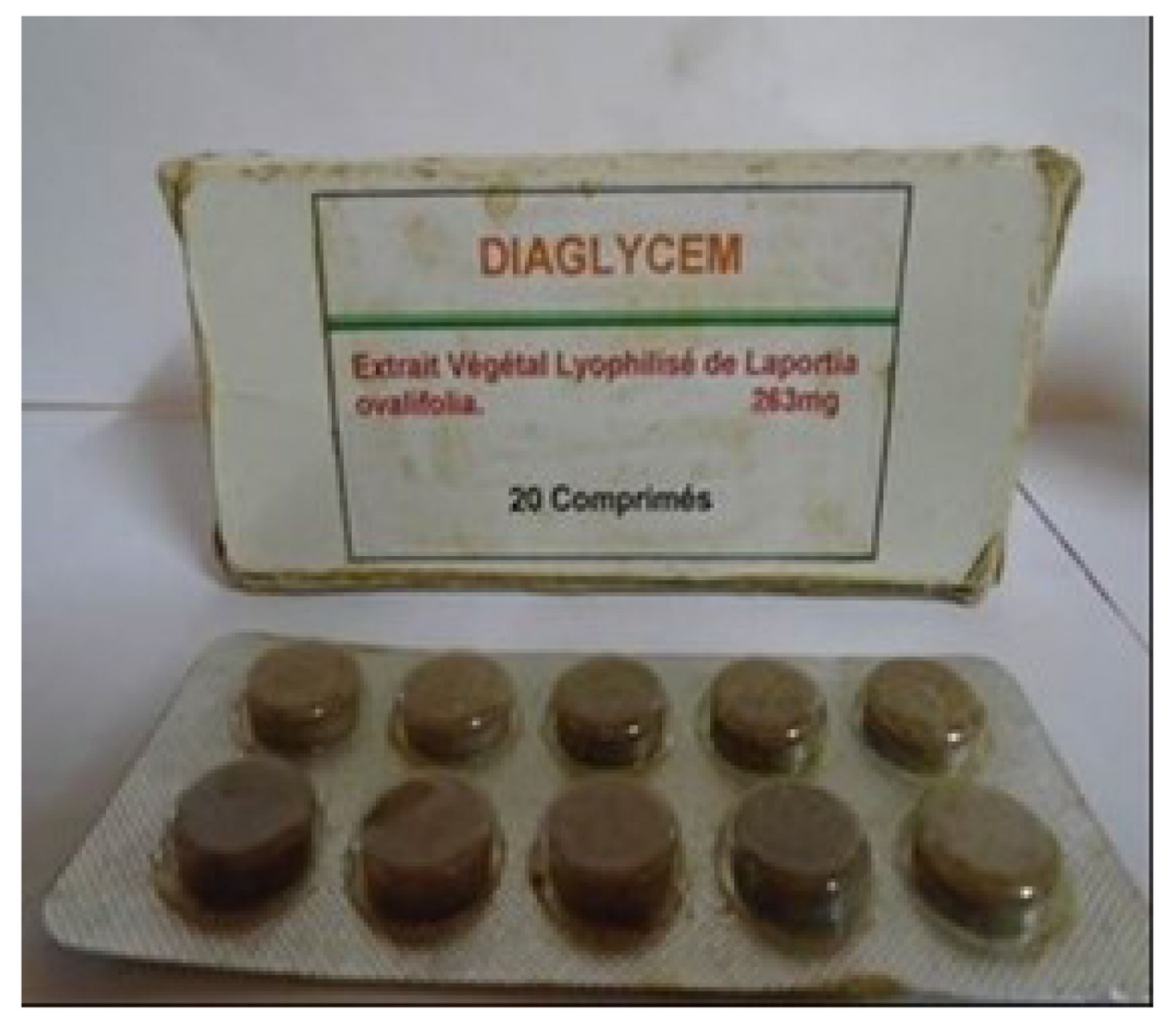
Capsules (This ITM was prepared in 2007 and this photograph was taken in June 2015 (no deterioration observed until now)).

**Figure 4:**
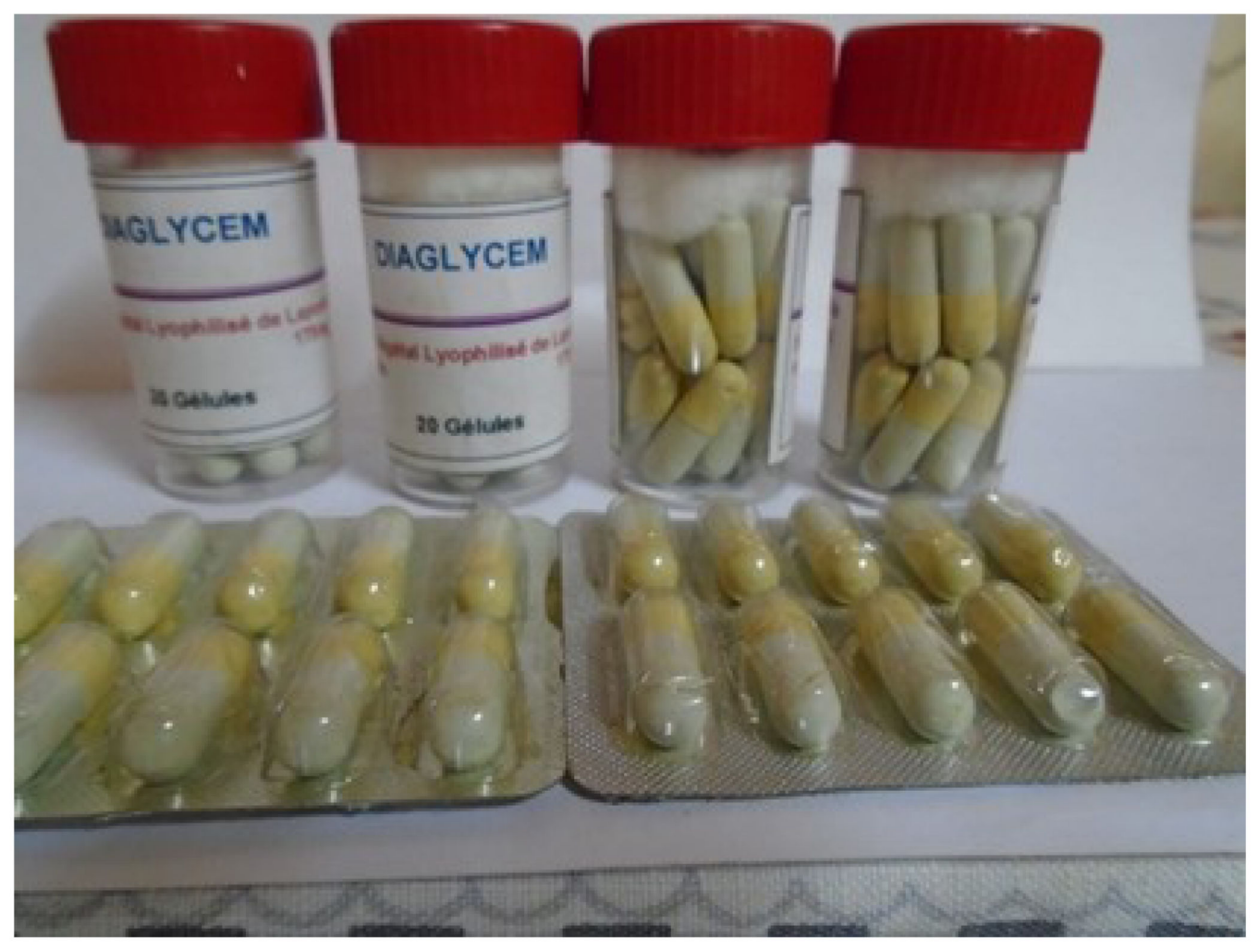
Capsules (This ITM was prepared in 2007 and this Photograph was taken in June 2015 (no deterioration observed until now)).

The aseptic conditions and the good conservation of the ITM are the guarantee of security of the tablets, the capsules, the oral suspension and the powder in sachets (Figures 1-4). The required instruments are easily accessible, and availability of plant materials make it possible to sustain the mass production of our ITM.

## Conclusion

Our results support the hypothesis that, enhanced approach of ITM’s preparation, can increase the conservation duration of traditional medicine. Consequently, nine years after the preparation of the four forms of an oral hypoglycemic improved traditional medicine seems to be efficient. The following improvements of *Laportea ovalifolia* traditional hypoglycemic recipe were conducted: the verification of the high blood glucose rate decreasing by following 30 non complicated diabetics, previously diagnosed in Fongo-Tongo health center and treated by a traditional healer; the formalization of the dose; the improvement of the hygiene conditions and certainly the overtaking of the usual conservation duration. The simplification of the standard approach of pharmaceutical preparation of medicines and the extraction by water make the originality of this new approach. Finally the availability of *Laportea ovalifolia* in its habitats that cover all the forestry areas until the altitude of 2800 m and its rapid growth are a natural asset for this ITM preparation.

## Figures and Tables

**Table 1:** Control of glycaemia in fasting diabetic patients treated at a traditional healer.

Diabetics	Glycaemia in fasting (g/l)		
Number	At the beginning of treatment	At the end of treatment	Reduction in blood glucose (%)
1	1.46	0.68	53.42
2	2.75	0.71	74.18
3	1.75	0.80	54.28
4	2.68	0.69	74.25
5	1.97	0.71	63.95
6	2.68	0.69	74.25
7	1.97	0.71	63.95
8	1.97	0.80	59.39
9	3.12	0.71	71.24
10	1.75	0.94	46.28
11	2.68	0.69	74.25
12	1.97	0.71	63.95
13	2.68	0.96	64.17
14	1.97	0.71	63.95
15	1.97	0.80	59.39
16	3.12	0.71	71.24
17	1.92	0.67	65.10
18	2.22	0.86	61.26
19	3.62	0.79	78.17
20	1.89	0.76	59.78
21	2.13	0.92	56.80
22	3.03	0.68	77.55
23	2.60	0.71	72.69
24	2.70	1.00	62.96
25	2.52	0.79	68.65
26	1.94	0.76	60.82
27	1.87	0.68	63.63
28	1.99	0.75	62.31
29	1.87	1.74	6.95
30	2.15	0.89	54.78
31	2.30	0.77	66.52
